# Differential effects of tissue inhibitor of metalloproteinase (TIMP)-1 and TIMP-2 on atherosclerosis and monocyte/macrophage invasion

**DOI:** 10.1093/cvr/cvv268

**Published:** 2015-12-08

**Authors:** Karina Di Gregoli, Sarah J. George, Christopher L. Jackson, Andrew C. Newby, Jason L. Johnson

**Affiliations:** School of Clinical Sciences, University of Bristol, Level 7, Bristol Royal Infirmary, Bristol BS2 8HW, UK

**Keywords:** Atherosclerosis, Monocyte, Macrophage, Matrix metalloproteinases, Plaque progression

## Abstract

**Aims:**

MMPs contribute to atherosclerotic plaque progression and instability, but the relative potency of their endogenous tissue inhibitors of metalloproteinases (TIMPs) as protective factors has not been defined. We therefore investigated the impact of TIMP-1 and TIMP-2 knockout on atherosclerotic plaque burden and composition in apolipoprotein E-knockout (*Apoe*^−/−^) mice and studied the underlying effects on monocyte/macrophage behaviour.

**Methods and results:**

Analysis of brachiocephalic artery plaques revealed comparable atherosclerotic lesion areas between *TIMP-1^−/−^ Apoe^−/−^* or *TIMP-2^−/−^ Apoe^−/−^* double deficient mice and relevant age-matched, strain-matched *Apoe^−/−^* controls after 8 weeks of high-fat feeding. However, lesions from *TIMP-2^−/−^ Apoe^−/−^* mice had higher levels of markers associated with plaque vulnerability, including increased macrophage: vascular smooth muscle cell ratios, larger necrotic core areas, reduced collagen contents, increased macrophage proliferation, and apoptosis frequencies, compared with *TIMP-1^−/−^Apoe^−/−^* and controls. In contrast, *TIMP-1^−/−^ Apoe^−/−^* animals only had a significant reduction in vascular smooth muscle cell content compared with *Apoe^−/−^* controls. *In vitro* and *in vivo* findings implicated heightened monocyte/macrophage invasion in the detrimental effects observed on atherosclerotic plaque composition in *TIMP-2^−/−^ Apoe^−/−^* mice. Moreover, TIMP-2 specifically decreased MMP-14-dependent monocyte/macrophage infiltration into sites of experimentally induced inflammation and established atherosclerotic lesions.

**Conclusion:**

Our data demonstrate that TIMP-2 plays a greater protective role than TIMP-1 during the pathogenesis of atherosclerosis, in part by suppressing MMP-14-dependent monocyte/macrophage accumulation into plaques.

## 1. Introduction

Atherosclerotic plaque instability underlies the majority of myocardial infarctions, most commonly through rupture of the fibrous cap.^1^ Plaque rupture results from excess hydrodynamic stress on a cap weakened by breakdown of the extracellular matrix (ECM).^2^ Such plaques are histologically characterized by large necrotic cores, a high macrophage content in their fibrous caps, and a concomitant reduction in collagen and smooth muscle cell density.^2^ In atherosclerotic plaques that are vulnerable to rupture, matrix degradation prevails, particularly at the macrophage-rich shoulder regions where secretion of several MMPs is elevated while their endogenous tissue inhibitors (TIMPs) are not, resulting in heightened proteolytic activity.^3,4^

Recruitment of fresh monocytes at sites distinguished as athero-susceptible is one of the critical events in lesion formation and plaque progression, although intra-plaque macrophage proliferation may also play a prominent role in advanced lesions.^5^ Circulating monocytes display heterogeneity and can be phenotypically separated into at least two distinct subsets in mice: ‘inflammatory’ monocytes are characterized by Ly6C^hi^ CCR2^hi^ CX3CR1^lo^ cell-surface expression, whereas ‘patrolling’ monocytes exhibit a Ly6C^lo^ CCR2^lo^ CX3CR1^hi^ profile.^6^ In man, monocyte expression of CD14 and CD16 is used to delineate similar subsets.^6^ It has been postulated that the divergent monocyte populations utilize proteases such as MMPs to permit their accumulation at sites of inflammation including atherosclerotic plaques.^7^ Indeed, MMP-12 and MMP-14 have both been demonstrated *in vitro* to regulate monocyte/macrophage transmigration and invasion.^8–11^ MMP-14, known also as membrane type-1 MMP (MT1-MMP), can direct peri-cellular matrix degradation due to its membrane localization in an active form.^12^ MMP-14 is also expressed by macrophages and foam-cell macrophages within human atherosclerotic plaques^13^ and has been implicated in acute myocardial infarction.^14^ Furthermore, loss- or gain-of-function studies imply an important role for MMP-14 in plaque stability.^15,16^ Consequently, MMP inhibitors could prove beneficial in reversing the detrimental effects of MMP-14.

As their name suggests, TIMPs, which are expressed by numerous vascular cell types within atherosclerotic plaques,^17^ tightly regulate endogenous MMP activity. However, the prolonged presence of net MMP activity in advanced plaques^18,19^ suggests that endogenous levels of TIMPs are inadequate to achieve complete inhibition. Intriguingly, TIMP-2 has been shown to inhibit MMP-14 activity, whereas TIMP-1 is less effective.^20^ Furthermore, overexpression studies have suggested that TIMP-2 may retard monocyte/macrophage invasion and therefore protect from plaque progression.^21^ Our current study aimed to compare the roles of TIMP-1 and TIMP-2 in atherosclerotic plaque progression using knockout models, particularly focusing on their effects on monocyte and macrophage invasion. We present novel data showing that TIMP-2 but not TIMP-1 acts as an important modulator of the MMP-14-directed monocyte/macrophage invasion that reduces accumulation in atherosclerotic lesions *in vivo* and therefore plays a protective role during the pathogenesis of atherosclerosis. Our findings indicate that MMP-14 is a pertinent therapeutic target for the prevention of clinical atherosclerosis and other inflammatory diseases. Furthermore, promoting TIMP-2 expression may represent an effective strategy to inhibit MMP-14.

## 2. Methods

### 2.1 Animals

Male and female mice homozygous null for the *Apoe* gene on a 71% C57BL/6J, 29% 129/SvJ background, were derived from a closed outbred colony housed in the Animal Unit of the University of Bristol. Previously generated *TIMP-1^−/−^*^22^ (background strain C57BL/6J; C57BL/6N) and *TIMP-2^−/−^*^23^ (background strain C57BL/6J) mice were kindly provided by Dr R Lijnen (University of Leuven, Leuven, Belgium). *Apoe^−/−^* mice were crossed with TIMP knockouts to generate *TIMP-1^−/−^ Apoe^−/−^* and *TIMP-2^−/−^ Apoe^−/−^* double knockout mice as well as their relevant age-, strain-, and sex-matched *Apoe^−/−^* single knockout littermate controls. Genetic fingerprinting of tail tip DNA revealed the strain background of the breeding colonies was 69.7% C57BL/6J, 30.3% 129/SvJ for *TIMP-1^+/−^ Apoe^+/−^*, and (71.6% C57BL/6J, 28.4% 129/SvJ) for *TIMP-2^+/−^ Apoe^+/−^* animals, C57BL/6N strain was not detected. Genomic DNA was extracted from tail tips for genotyping by PCR.

The housing and care of the animals and all the procedures used in these studies were performed in accordance with the ethical guidelines and regulations of the University of Bristol and the UK Home Office. The investigation conforms with the Guide for the Care and Use of Laboratory Animals published by the US National Institutes of Health (NIH Publication No. 85–23, Eighth Edition, revised 2011). To evaluate any effect of TIMP gene knockout on normal vessel development, subsets of animals were fed standard chow diet from weaning for 6 weeks and then terminated. To evaluate atherosclerosis, animals of 8 weeks of age were fed high-fat diet containing 21% (wt : wt) pork lard and supplemented with 0.15% (wt : wt) cholesterol (Special Diet Services, Witham, UK) for 8 weeks. To ensure that the studies were adequately powered, group sizes in excess of 20 animals were used.

Animals were anaesthetized by intraperitoneal injection of sodium pentobarbitone (500 mg/kg of bodyweight) before exsanguination by perfusion via the abdominal aorta with PBS at a constant pressure of 100 mmHg, with outflow through the incised jugular veins. This was followed by constant pressure perfusion with 10% formalin.

### 2.2 Plasma lipid profile

Plasma cholesterol levels were quantified as previously described,^24^ using the BioVison Cholesterol Assay Kit (Cambridge BioSciences, Cambridge, UK).

### 2.3 Histology, plaque morphometrics, and histological analyses

Sections (3 µm) were cut from the atherosclerosis-prone areas of brachiocephalic arteries and the aortic root as previously described.^9^ Sections were subjected to immunohistochemical and morphometric analysis (see Expanded Methods online).

### 2.4 *In vitro* studies on monocyte/macrophages

Freshly isolated mouse monocytes or 7-day M-CSF-differentiated macrophages were assessed by Q-PCR, flow cytometry, and immunocytochemistry for MMP-14 and monocyte/macrophage subset markers (see Expanded Methods online). Gelatinolytic activity was assessed in cultured mouse monocytes by *in situ* zymography, as described previously.^25^

### 2.5 *In vitro* invasion assay

Monocyte/macrophage invasion *in vitro* was assessed using Matrigel™-coated transwell inserts (Merck Millipore, Watford, UK) as described previously.^25^ Transwell inserts containing 8 μm pore membranes were coated with 25 μL/well of Matrigel™ (BD Biosciences, Oxford, UK). Fifteen nanograms per millilitre of MMP-14 blocking antibody (BAb) (Millipore), purified mouse recombinant-TIMP2 (10 µM) (Calbiochem), or mouse IgG (15 ng/mL) was added to the Matrigel of the appropriate inserts. Monocyte/macrophages were added to the upper portion of the transwell. RPMI/FCS (600 μL) supplemented with 30 ng/mL of mouse recombinant monocyte chemoattractant protein-1 (MCP-1) and 30 ng/mL of mouse recombinant Fractalkine (CX3CL1) (R&D System, Abingdon, UK) was placed in the lower wells to induce transmigration/invasion. After 48 h, the number of migrated/invaded cells were quantified and expressed as a percentage of total cells.

### 2.6 *In vivo* monocyte/macrophage invasion assay

TIMP-2 WT and KO mice were anaesthetised by inhalation with isofluorane and 2–6 Matrigel™ (BD Biosciences, Oxford, UK) infused 1 cm^3^ polyurethane sponges (Baxter Scientific, Newbury, Berkshire, UK) placed under the dorsal skin for 11 days, and mice fed normal diet to allow the accumulation of monocyte/macrophages as previously described.^9^ After 11 days, the sponges were retrieved and fixed in 10% formalin, then subjected to flow cytometry or immunohistochemistry for cell markers as deemed necessary.

### 2.7 *In vivo* experiment for monocyte recruitment into atherosclerotic lesions

*Apoe^−/−^* mice (8 weeks of age, *n* = 22) were fed a high-fat diet containing 21% (wt : wt) pork lard and supplemented with 0.15% (wt : wt) cholesterol (Special Diet Services, Witham, UK) for 8 weeks. To assess monocyte/macrophage recruitment to sites of inflammation, 8 of the 16 high-fat fed mice, termed here after as ‘recipient’ mice, had three 1 cm^3^ polyurethane sponges subcutaneously implanted as described above and returned to a high-fat diet for a further 8 days. Of the remaining mice, after 9 weeks of high-fat feeding, monocytes were isolated by density gradient separation and adhesion on plastic from *n* = 6 and are here after referred to as ‘donor’ mice. Adherent monocytes were fluorescently labelled with Vybrant Carboxyfluorescein Diacetate, Succinimidyl Ester (CFDA SE) Cell Tracer Kit (Invitrogen, Life Technologies, Paisley, UK) according to manufacturer instructions. Monocytes in suspension from each preparation were split and then incubated with either mouse 15 μg/mL MMP-14 blocking antibody (Millipore, Watford, UK) or mouse IgG for 30 min at room temperature. Labelled monocytes (6.5 × 10^4^ per mouse) pre-treated with and without MMP-14 blocking antibody were injected into *Apoe^−/−^*-‘recipient’ mice via the tail vein (*n* = 16, of which *n* = 8 mice with subcutaneous sponges). Animals were terminated 60 h after cell injection and samples collected for subsequent analysis.

### 2.8 Statistical analysis

Values are expressed as mean ± S.E.M. Group values were compared using the computer program InStat (GraphPad Software, San Diego, CA, USA). For the comparison of group means, a check was first made for similar variances: if this was passed then an unpaired two-sample two-tailed Student's *t*-test was carried out. If the variances were significantly different, then an unpaired two-sample two-tailed *t*-test with Welch's correction was used. Statistical differences between monocyte/macrophages from the same preparation were analysed by Student’s paired *t*-test. For the comparison of multiple groups, an analysis of variance (ANOVA) test was used, and a Student–Newman–Keuls multiple comparisons *post hoc* test employed when statistical differences were detected. If a Bartlett's test revealed that the differences among the S.D. of groups were significant, a Kruskal–Wallis non-parametric ANOVA was used and a Dunn post-test applied if *P* < 0.05. Contingency data (presence of elastin breaks) were analysed by Fisher's exact test. In all cases, statistical significance was concluded where the two-tailed probability was <0.05.

## 3. Results

### 3.1 Effects of TIMP-1 or TIMP-2 deficiency on morphometry of the brachiocephalic artery in chow-fed animals

There were no significant differences in total vessel, medial, or luminal areas between brachiocephalic arteries of *TIMP-1^−/−^ Apoe^−/−^* or *TIMP-2^−/−^ Apoe^−/−^* double knockout mice compared with their respective strain-matched *Apoe^−/−^* controls, after 8 weeks of chow diet (see Supplementary material online, *Table S1*). These findings suggest that physiological development of the brachiocephalic artery is unaffected by deficiency of individual TIMPs.

### 3.2 TIMP-2 but not TIMP-1 deficiency aggravates markers of atherosclerotic plaque instability

**Figure 1:**
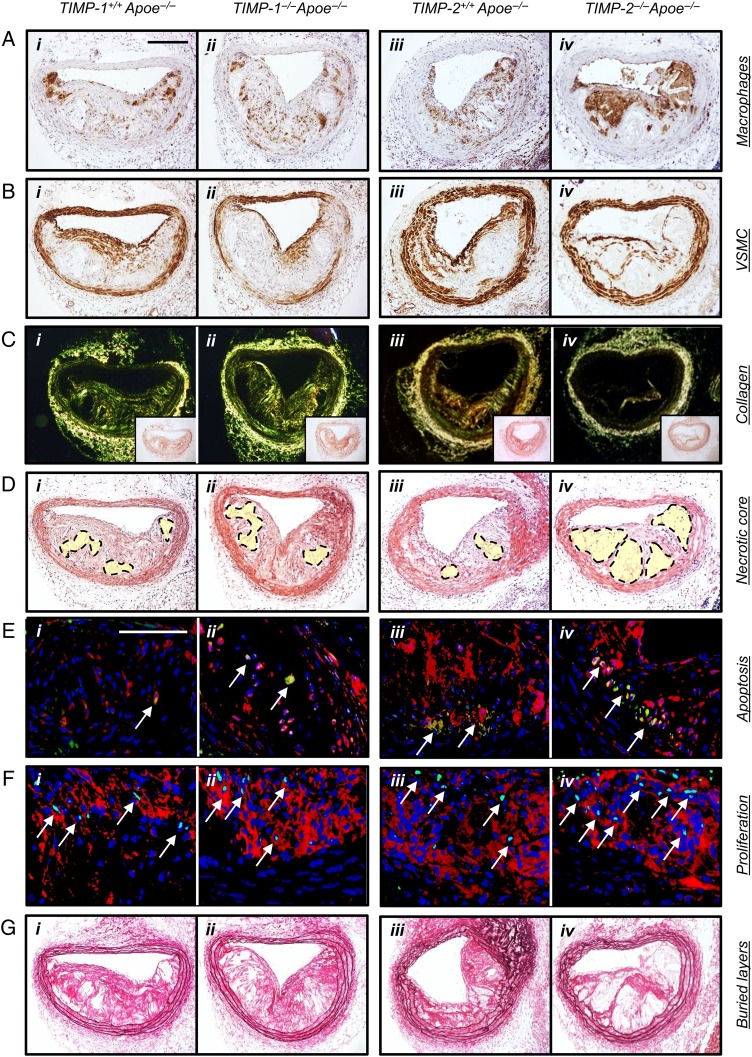
TIMP-2 deficiency retards brachiocephalic plaque progression and alters plaque composition. Representative images of histological sections of brachiocephalic atherosclerotic plaques from 8-week high-fat-fed *TIMP-1^+/+^ Apoe^−/−^ (i; n* = 24), *TIMP-1^−/−^ Apoe^−/−^* mice (*ii*; *n* = 24), *TIMP-2^+/+^ Apoe^−/−^ (iii; n* = 28), and *TIMP-2^−/−^ Apoe^−/−^* mice (*iv*; *n* = 29). Sections are histochemically or immuno-stained for CD68 (macrophages; *A*), α-smooth muscle actin (vascular smooth muscle cells, *B*), picrosirius red (collagen; *C*), haematoxylin and eosin (*D*), cleaved caspase-3 (green) and CD68 (red) (apoptosis; *E*), PCNA (green) and CD68 (red) (proliferation; *F*), or elastin van Gieson (*G*). Scale bar in A*i* represents 100 µm and is applicable to *A*–*D* and *G*. Scale bar in Ei represents 50 µm and is applicable to all panels in *E* and *F.* Insets in *C* (*i –iv*) are matching brightfield images of picrosirius red stained sections of the relevant panels. Areas depicted by dotted lines in *D* (*i–iv*) represent the relative necrotic core area assessed. Arrows in *E* and *F* highlight apoptotic and proliferating macrophages, respectively, while nuclei are counterstained with DAPI (blue).

**Figure 2:**
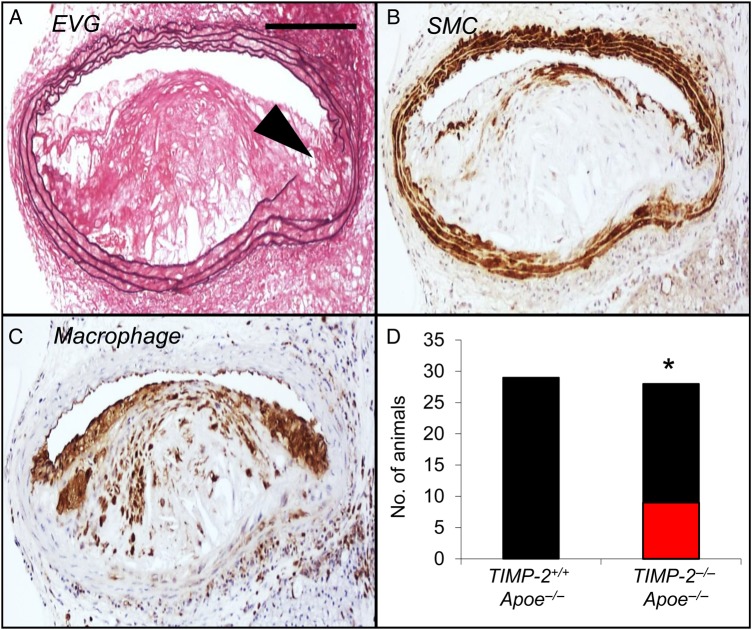
TIMP-2 deficiency induces elastin fragmentation. (*A*–*C*) Representative images of serial sections from an atherosclerotic brachiocephalic artery with a marked elastin fragmentation (black arrowhead in *A*) which have been immunohistochemical stained for (*A*) elastin van Gieson, (*B*) smooth muscle cells, or (*C*) macrophages from a *TIMP-2^−/−^ Apoe^−/−^* mouse. (*D*) Quantification of the number of *TIMP-2^+/+^ Apoe^−/−^* and *TIMP-2^−/−^ Apoe^−/−^* mice without (black bars) or with (red bars) elastin breaks (*P < 0.05; *n* = 27/28 per group; data expressed as mean ± SEM). Scale bar in *A* represents 100 µm and is applicable to all panels.

Immunohistochemistry of advanced brachiocephalic plaques from 8-week high-fat-fed mice revealed that 12.7 ± 4.1 and 14.9 ± 3.6% of macrophages expressed TIMP-1 or TIMP-2, respectively (see Supplementary material online, *Figure S1*). After 8-week high-fat feeding, no significant differences in plasma lipids were observed between *TIMP-1^−/−^ Apoe^−/−^* or *TIMP-2^−/−^ Apoe^−/−^* double knockout mice and their respective strain-matched *Apoe^−/−^* controls (see Supplementary material online, *Table S2*). No significant differences were observed in any of the measured parameters within the brachiocephalic arteries between *TIMP-1^+/+^ Apoe^−/−^* and *TIMP-2^+/+^ Apoe^−/−^* wild-type mice, except for buried layers, which were less evident in *TIMP-2^+/+^ Apoe^−/−^* animals (*Table 1*; see Supplementary material online, *Figure S2*). Direct comparison between *TIMP-1^−/−^ Apoe^−/−^* and *TIMP-2^−/−^ Apoe^−/−^* mice revealed no differences in plaque size or vascular smooth muscle cell content (*Table 1*; see Supplementary material online, *Figure S2* and *Figure 1*). However, plaques from both *TIMP-1^−/−^ Apoe^−/−^* and *TIMP-2^−/−^ Apoe^−/−^* mice demonstrated reduced vascular smooth muscle cell content compared with wild-type control animals (*P* < 0.01; *Table 1*; see Supplementary material online, *Figure S2* and *Figure 1*). Further analysis of brachiocephalic artery lesions from *TIMP-1^−/−^ Apoe^−/−^* mice revealed no significant changes in all other plaque composition parameters compared with *TIMP-1^+/+^ Apoe^−/−^* and *TIMP-2^+/+^ Apoe^−/−^* controls, respectively. Strikingly, TIMP-2 deficiency led to a dramatic shift in plaque composition compared with *TIMP-1^−/−^ Apoe^−/−^* (and both wild-type control mice; *Table 1*; see Supplementary material online, *Figure S2* and *Figure 1*). With regard to cellular content, plaques from *TIMP-2^−/−^ Apoe^−/−^* mice exhibited a 33% (*P* < 0.05) increase in macrophage number compared with *TIMP-1^−/−^ Apoe^−/−^* mice (*Table 1*; see Supplementary material online, *Figure S2* and *Figure 1*). Conversely, picrosirius red staining of fibrillar collagens and observation under polarized light revealed that plaque collagen content was decreased (66%; *P* < 0.01) in *TIMP-2^−/−^ Apoe^−/−^* mice, compared with *TIMP-1^−/−^ Apoe^−/−^* mice (*Table 1*; see Supplementary material online, *Figure S2* and *Figure 1*). A similar effect on collagen content was detected in comparison with *TIMP-2^+/+^ Apoe^−/−^* controls, presumably due to increased degradation as collagen production was unaltered between aortic smooth muscle cells from either *TIMP-2^+/+^* or *TIMP-2^−/−^* mice (see Supplementary material online, *Figure S3*), even after phenotypic switching with PDGF-BB and IL-1β (see Supplementary material online, *Figure S3*). We next evaluated the size of the plaque necrotic core by measuring the area of haematoxylin-negative, acellular areas in the intima^26^ and observed that TIMP-2 deficiency was associated with a significant increase in necrotic core size (47%; *P* < 0.01; *Table 1*; see Supplementary material online, *Figure S2* and *Figure 1*), compared with *TIMP-1^−/−^ Apoe^−/−^* mice. Consistently, we detected a significantly increased frequency of macrophages undergoing apoptosis in plaques from *TIMP-2^−/−^ Apoe^−/−^* mice relative to *TIMP-1^−/−^ Apoe^−/−^* animals (*P* < 0.01; *Table 1*; see Supplementary material online, *Figure S2* and *Figure 1*). However, because macrophage accumulation was increased in spite of the increased frequency of apoptosis, we also assessed proliferation indices. Indeed, heightened lesional macrophage proliferation has been proposed to drive murine atherosclerotic plaque progression.^5^ The number of macrophages assessed to be undergoing proliferation was increased within lesions of *TIMP-2^−/−^ Apoe^−/−^*-related *TIMP-1^−/−^ Apoe^−/−^* mice (100%; *P* < 0.01; *Table 1*; see Supplementary material online, *Figure S2* and *Figure 1*). Collectively, TIMP-2 deficiency resulted in alterations in plaque composition that have been previously taken as markers of increased plaque instability. Consistent with this, the lesion compositional changes translated to a heightened plaque vulnerability index^27^ in mice lacking TIMP-2, compared alongside TIMP-1-deficient mice and wild-type control animals (*Table 1*; see Supplementary material online, *Figure S2*). We additionally counted buried fibrous layers (VSMC-rich layers invested with elastin that overlay foam-cell macrophages), which have been postulated as another surrogate marker of plaque vulnerability in mice^24^ and humans.^28^ Our analysis revealed no difference between *TIMP-1^−/−^ Apoe^−/−^* and *TIMP-2^−/−^ Apoe^−/−^* mice, although a significant 2.3-fold increase in the number of plaques containing buried fibrous layers from *TIMP-2^−/−^ Apoe^−/−^* compared with *TIMP-2^−/−^ Apoe^−/−^* mice was observed (*Table 1*; see Supplementary material online, *Figure S2* and *Figure 1*). Similarly, no alterations in evidence of elastin fragmentation was seen between *TIMP-1^−/−^ Apoe^−/−^* and *TIMP-2^−/−^ Apoe^−/−^* mice, whereas more prevalent elastin fragmentation was detected in both the atherosclerotic brachiocephalic arteries (*Table 1*; see Supplementary material online, *Figure S2* and *Figure 2*) and at the aortic root (see Supplementary material online, *Figure S4*) of *TIMP-2^−/−^ Apoe^−/−^* mice compared with *TIMP-2^−/−^ Apoe^−/−^* mice animals.

**Table 1:** Effects of TIMP-1 or TIMP-2 deficiency on Apoe^−/−^ mouse plaque area and characteristics after 8-week high-fat feeding (*n* = 24–29/group)

	TIMP-1^+/+^ Apoe^−/−^	TIMP-1^−/−^ Apoe^−/−^	TIMP-2^+/+^ Apoe^−/−^	TIMP-2^−/−^ Apoe^−/−^
Lesion area ×10^3^ µm^2^ (Brachiocephalic)	89 ± 10	69 ± 9	74 ± 9	67 ± 9
Macrophage (%)	16.8 ± 3.6	28.4 ± 10.5	21.6 ± 3.1	37.8 ± 4.0^a,b,c^
VSMC (%)	20.6 ± 3.2	7.6 ± 1.2^a,b^	16.8 ± 1.7	9.0 ± 1.2^a,b^
Collagen (%)	19.9 ± 4.0	24.2 ± 4.9	15.4 ± 2.3	8.3 ± 1.5^a,b,c^
Necrotic core (%)	19.3 ± 2.6	24.7 ± 2.7	18.9 ± 3.7	36.4 ± 3.4^a,b,c^
Apoptosis (%)	10.0 ± 3.6	13.6 ± 2.9	21.4 ± 3.8	35.8 ± 4.2^a,b,c^
Proliferation (%)	20.1 ± 5.1	25.1 ± 6.1	34.6 ± 4.1	50.3 ± 5.1^a,b,c^
Vulnerability index	1.1 ± 0.2	0.6 ± 0.2	0.8 ± 0.1	0.2 ± 0.1^a,b,c^
Buried layers	0.9 ± 0.2	0.7 ± 0.2	0.3 ± 0.1^a^	0.8 ± 0.1^b^
Elastin breaks	1/24	5/24	0/29	9/28^a,b^

^a^Significant difference (*P* < 0.05) from TIMP-1^+/+^ Apoe^−/−^ mice.

^b^Significant difference (*P* < 0.05) from TIMP-2^+/+^ Apoe^−/−^ mice.

^c^Significant difference (*P* < 0.05) from TIMP-1^−/−^ Apoe^−/−^ mice.

### 3.3 TIMP-2 but not TIMP-1 deficiency retards monocyte/macrophage invasion

**Figure 3:**
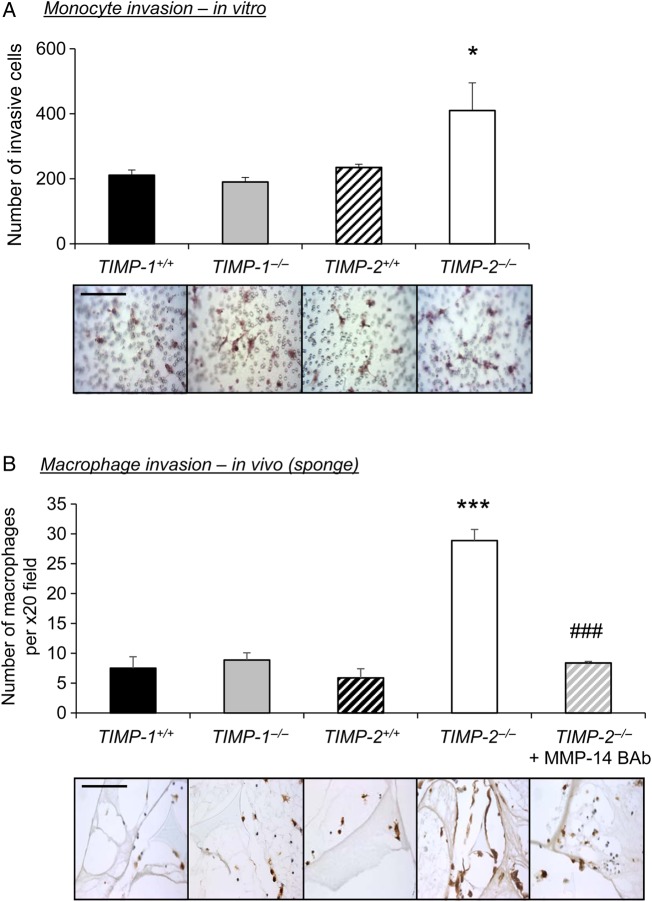
TIMP-2 deficiency retards monocyte/macrophage invasion. (*A*) Representative CD11b IHC-labelled Matrigel-coated invasion inserts and quantification of *TIMP-1^+/+^*, *TIMP-1^−/−^*, *TIMP-2^+/+^* and *TIMP-2^−/−^* monocyte invasion *in vitro* (*n* = 4; *P < 0.05 denotes significant difference from all other groups). Scale bar represents 50 µm and is applicable to all panels. (*B*) Representative CD11b IHC-labelled images and quantification of macrophage number within subcutaneous sponges from *TIMP-1^+/+^*, *TIMP-1^−/−^*, *TIMP-2^+/+^* and *TIMP-2^−/−^* mice, and *TIMP-2^−/−^* mice with sponges containing an MMP-14-blocking antibody (BAb; 15 ng/mL) (*n* = 6 per group; ***P < 0.001 denotes significant difference from all groups; ^###^*P* < 0.001 denotes significant difference from *TIMP-2^−/−^* mice). Scale bar represents 50 µm and is applicable to all panels.

Due to the striking differences in macrophage accumulation within plaques of mice deficient in TIMP-2, as opposed to TIMP-1 knockout animals, compared with their relevant wild-type controls (*Table 1* and *Figure 1*), we assessed the roles of TIMP-1 and TIMP-2 on monocyte invasion *in vitro* and *in vivo*. Accordingly, monocyte invasion through a Matrigel-coated transwell chamber in response to MCP-1 and fractalkine-induced chemo-attraction^21^ was significantly increased by monocytes from *TIMP-2^−/−^* mice showed more invasion *in vitro* when compared with *TIMP-2^+/+^* wild-type cells (2-fold increase; *P*≤ 0.05; *Figure 3A*). In contrast, invasion of *TIMP-1^−/−^* monocytes was comparable to *TIMP-1^+/+^* wild-type controls (*Figure 3A*). Furthermore and consistent with our *in vitro* data, the number of monocyte/macrophages recruited into Matrigel-infused sponges was unaffected between *TIMP-1^−/−^* and *TIMP-1^+/+^* wild-type control mice (*Figure 3B*), whereas significantly increased monocyte/macrophage accumulation was observed in *TIMP-2^−/−^* mice compared with *TIMP-2^+/+^* wild-type controls (4.3-fold; *P* < 0.001, *Figure 3B*). These data support a key role for TIMP-2 in arresting monocyte invasion and may explain the observed increase in macrophage numbers within brachiocephalic plaques of *TIMP-2^−/−^* mice.

### 3.4 MMP-14 is expressed by CX3CR1^hi^/CCR2^lo^/Ly6C^lo^ monocytes but is rapidly up-regulated in all monocytes after adhesion

**Figure 4:**
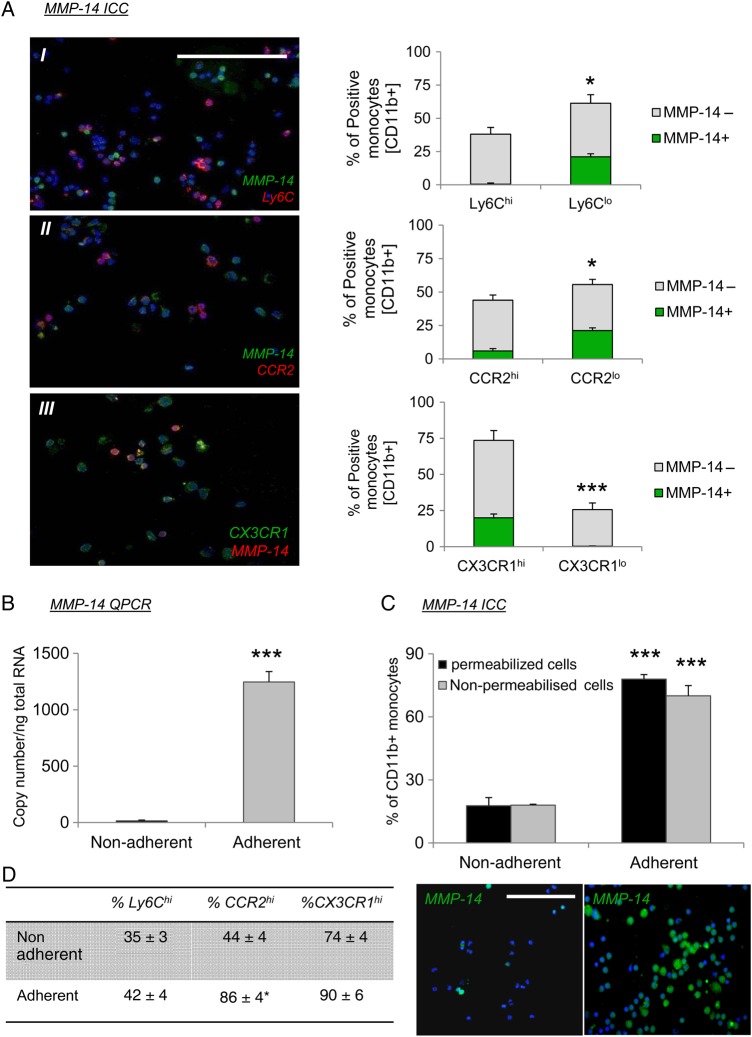
MMP-14 is expressed by Ly6C^lo^ monocytes but is rapidly up-regulated in all monocytes after adhesion. (*A*) Co-labelling and quantification of MMP-14 with (*I*) Ly6C, (*II*) CCR2, and (*III*) CX3CR1 in CD11b positively selected monocytes. Grey bars in graphs represent MMP-14-negative monocytes while green bars signify MMP-14 positive cells (*denotes *P* < 0.05 and ***denotes *P* < 0.001; *n* = 6). Scale bar represents 50 µm and is applicable to all panels. (*B* and *C*) QPCR for MMP-14 mRNA expression (*B*) and representative immunohistochemical labelling and quantification of MMP-14 protein expression, in non-adherent and adherent monocytes (*n* = 4; ***P < 0.001 and represents significant difference from non-adherent monocytes). Scale bar represents 50 µm and is applicable to both panels. (*D*) Quantification of non-adherent and adherent monocyte Ly6C, CCR2, and CX3CR1 expression (*n* = 4; *P < 0.05).

We have previously demonstrated that systemic adenoviral-mediated overexpression of TIMP-2, but not TIMP-1, can modulate intra-plaque gelatinolytic activity.^21^ In addition, exogenous TIMP-2 retarded *in vitro* monocyte/macrophage invasion, whereas TIMP-1 was ineffective.^21^ Taken together with our current findings, these results imply that TIMP-1 and TIMP-2 exert disparate inhibitory effects. It is known that TIMP-1 poorly inhibits type I MT-MMPs, such as MMP-14, −15, −16, and −24, but are effectively inhibited by TIMP-2.^20^ Accordingly, we assessed the expression of the four type I MT-MMPs in monocytes and mature macrophages. QPCR analysis revealed that MMP-14 is significantly more abundant in monocytes and macrophages than other type I MT-MMPs (see Supplementary material online, *Figure S5A*). Similarly, a greater number of macrophages within the shoulder regions of mouse brachiocephalic plaques expressed MMP-14 than MMP-15, −16, or −24 (see Supplementary material online, *Figure S5B*), implying a prominent role for MMP-14 in monocyte/macrophage accumulation. Indeed, MMP-14 has been directly implicated in the migration/invasion of monocytes through the endothelium.^10^ We therefore assessed whether TIMP-2-dependent MMP-14 activity regulated the migration/invasion of monocyte/macrophages *in vitro* and *in vivo*, and therefore underlies the observed detrimental effects on atherosclerosis observed in *TIMP-2^−/−^ Apoe^−/−^* mice. In the first instance, we measured MMP-14 expression in circulating and adherent monocytes. Immunocytochemical and flow cytometry analysis showed that MMP-14 is expressed in only 18 ± 4% of circulating non-adherent monocytes (*Figure 4A* and see Supplementary material online, *Figure S6A*). Dual labelling with recognized markers of well-characterized murine monocyte subsets (Ly6C, CCR2, and CX3CR1) revealed that MMP-14 expression is not associated with Ly6C- or CCR2-positive monocytes (1 ± 0.8% MMP-14^+^Ly6C^+^ and 6 ± 2% MMP-14^+^CCR2^+^ double positive cells, respectively; *P* < 0.05; *Figure 4A*). Conversely, the majority of MMP-14 positive monocytes co-expressed CX3CR1 (99.7 ± 0.2% MMP-14^+^CX3CR1^+^; *P* < 0.001; *Figure 4A* and see Supplementary material online, *Figure S6B*). These findings imply that MMP-14 serves as an additional marker of the LyC6^lo^ CCR2^lo^ CX3CR1^hi^ monocyte subset that performs a patrolling role in early responses to cell injury and infection^29^ and also in tissue repair after ischaemic injury.^30^ Interestingly, upon adhesion to plastic MMP-14, mRNA was significantly up-regulated (*P* ≤ 0.05; *Figure 4B*) and consequently the percentage of MMP-14 positive cells was increased 4.4-fold (*P* ≤ 0.001) compared with non-adherent cells (*Figure 4C*). Moreover, no difference in monocyte MMP-14 immuno-positivity was identified between non-permeabilized and permeabilized cells (*Figure 4C*), implying that MMP-14 is localized to the cell membrane ready to facilitate cell invasion.^11^ Finally, the percentage of CCR2 positive monocytes after cell adhesion was significantly increased by 1.9-fold (*P* < 0.01; *Figure 4D*) to a comparable level of CX3CR1, indicating that upon adhesion, monocytes respond to MCP-1 and fractalkine chemo-attraction, irrespective of Ly6C expression.

### 3.5 MMP-14 promotes and TIMP-2 retards monocyte invasion and recruitment *in vitro* and *in vivo*

**Figure 5:**
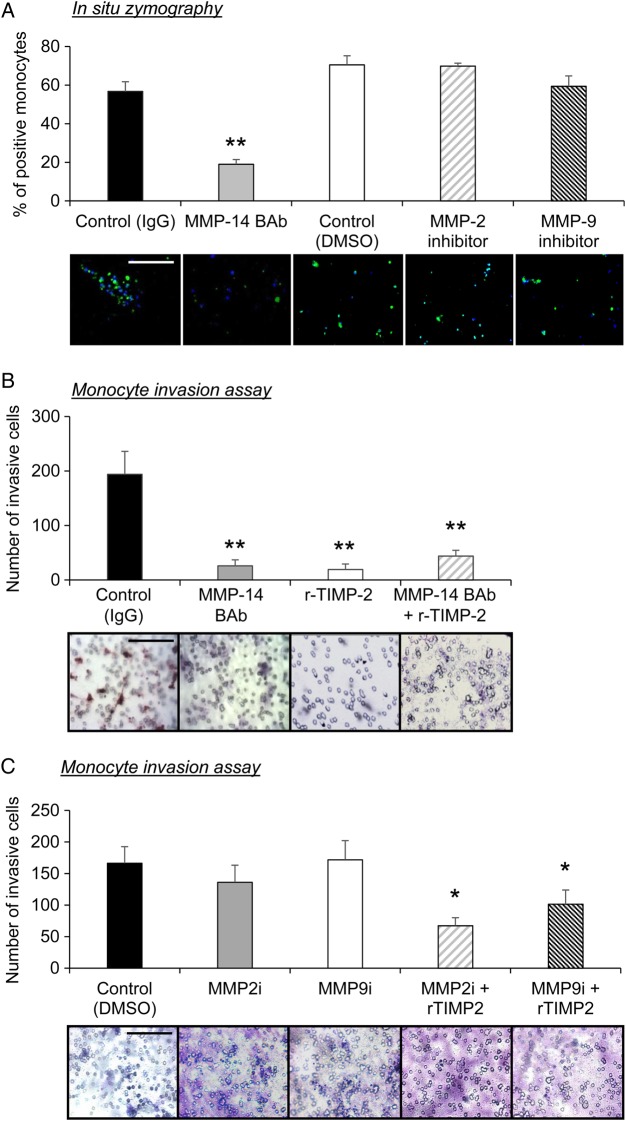
TIMP-2 retards MMP-14-dependent monocyte invasion *in vitro*. (*A*) Quantification of gelatinolytic activity, as assessed by *in situ* zymography of monocytes incubated with substrate plus goat IgG (control), 15 ng/mL MMP-14 blocking antibody (BAb), DMSO (control), MMP-2 inhibitor. (MMP-2i; 1 nM), or MMP-9 inhibitor. (MMP-9i; 5 nM) Green fluorescence represents gelatinolytic activity (**P < 0.01 and denotes significant difference from IgG control; *n* = 6 per group; data expressed as mean ± SEM). (*B*) Representative Matrigel-coated invasion inserts and quantification of monocyte invasion with and without an MMP-14 blocking antibody, (BAb; 15 ng/mL), recombinant TIMP-2 (5 nM), or both, and incubated for 48 h (*n* = 4; **P < 0.01 denotes significant difference from IgG control). (*C*) Representative Matrigel-coated invasion inserts and quantification of monocyte invasion with and without MMP-2 inhibitor (MMP-2i; 1 nM), or MMP-9 inhibitor (MMP-9i; 5 nM), plus recombinant TIMP-2 (5 nM), and incubated for 48 h (*n* = 4; *P < 0.05 denotes significant difference from IgG control, MMP-2i and MMP-9i). Scale bar represents 50 µm and is applicable to all panels.

To test our hypothesis that increased MMP-14 expression endows monocyte/macrophages with an enhanced migratory capacity, we measured MMP-14 activity and performed *in vitro* migration assays. *In situ* zymography revealed that adherent monocytes exhibited abundant MMP-14-dependent gelatinolytic activity, which could be significantly reduced by co-incubation with an MMP-14-neutralizing antibody, when compared alongside IgG-treated control cells (three-fold decrease; *P* ≤ 0.01; *Figure 5A*). MMP-2 and MMP-9 are also potent gelatinases; however co-incubation with inhibitors to either of these MMPs failed to diminish the gelatinolytic activity observed in control monocytes (*Figure 5A*). Monocyte invasion through a Matrigel-coated transwell chamber in response to MCP-1 and fractalkine-induced chemo-attraction^21^ was significantly retarded in the presence of an MMP-14-neutralizing antibody (91 ± 4% reduction; *P* ≤ 0.01; *Figure 5B*), mouse recombinant TIMP-2 (81 ± 10% reduction; *P* ≤ 0.01; *Figure 5B*), or when given in combination (77 ± 8% reduction; *P* ≤ 0.01; *Figure 5B*). Conversely treatment with either an MMP-2 or MMP-9 inhibitor failed to retard monocyte invasion compared with exogenous TIMP-2 (*Figure 5C*). These data suggest that inhibition of monocyte MMP-14 activity by endogenous TIMP-2 retards their invasion and may explain the observed increase in macrophage numbers within brachiocephalic plaques of *TIMP-2^−/−^* mice.

**Figure 6:**
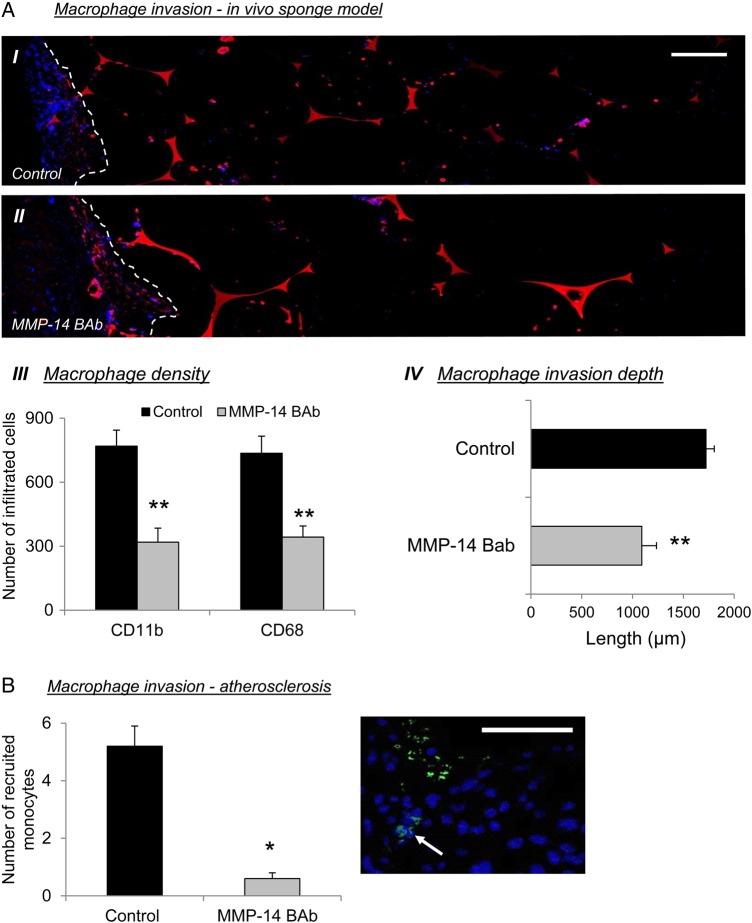
TIMP-2 retards MMP-14-dependent monocyte invasion *in vivo*. (*A*) Representative CD11b IHC-labelled images (*I* and *II*) and quantification of macrophage density (*III*) and invasion depth (*IV*), in an *in vivo* subcutaneous sponge invasion assay, in the presence of an MMP-14-blocking antibody (BAb; 15 ng/mL) compared with IgG controls (dotted line represents edge of sponge; *n* = 6 per group; **P < 0.01 denotes significant difference from IgG control-infused sponges). Scale bar represents 50 µm and is applicable to both panels. (*B*) Assessment of fluorescently labelled monocytes pre-treated with or without an MMP-14-blocking antibody (BAb; 15 ng/mL), in established atherosclerotic lesions (*n* = 6 per group; *P < 0.05 denotes significant difference from IgG control; white arrows indicate labelled monocytes). Scale bar represents 50 µm.

To extend our findings into an *in vivo* model, we examined the effect of MMP-14 inhibition on monocyte/macrophage recruitment in a subcutaneous granuloma invasion assay.^9^ In line with our *in vitro* observations, the number of monocyte/macrophages present in Matrigel-infused subcutaneous sponges was significantly reduced by 2.4-fold (*P* ≤ 0.001) and 2.1-fold (*P* ≤ 0.002), respectively, as assessed by the number of CD11b- or CD68-positive nucleated cells, in the presence of an MMP-14-neutralizing antibody compared with IgG control sponges (*Figure 6A*). Furthermore, MMP-14 inhibition significantly reduced the average distance of monocyte/macrophage invasion into sponges (1.6-fold decrease; *P* ≤ 0.001; *Figure 6A*) compared with controls. We also assessed the potential contributory role of altered intra-sponge monocyte/macrophage proliferation and/or apoptosis to the observed reduction in cell accumulation. However, using validated markers (cleaved caspase-3 and PCNA), apoptosis or proliferation markers were negligible in monocyte/macrophages of sponges in the presence of an MMP-14-neutralizing antibody or IgG controls (data not shown). Additionally, circulating monocyte numbers were not altered between mice receiving either MMP-14-neutralizing antibody or IgG control-infused sponges (see Supplementary material online, *Figure S7A*). Taken together, these findings strongly imply that MMP-14 directly permits monocyte/macrophage invasion *in vivo*.

To corroborate and relate our findings to atherosclerosis, we investigated whether nullifying MMP-14 impedes monocyte recruitment into plaques. Accordingly, we performed an *in vivo* adoptive transfer experiment (using a previously described method^31^). We intravenously injected hypercholesterolaemic *Apoe^−/−^* mice harbouring pre-existing atherosclerotic plaques with fluorescently labelled monocytes retrieved from the circulation of donor mice. Monocytes were pre-incubated or not with an MMP-14-neutralizing antibody. Recipient mice showed no apparent adverse effects, and we isolated brachiocephalic arteries 60 h after the injections. We analysed histological sections of the proximal brachiocephalic artery with light and fluorescent microscopy for labelled macrophages that were adherent to or within the atherosclerotic plaques. Consistent with the data from the *in vitro* invasion assays and *in vivo* sponge experiments, the number of fluorescent monocytes recruited into brachiocephalic artery plaques was significantly reduced 8.7-fold (*P* ≤ 0.01; *Figure 6B*) when MMP-14 activity was neutralized. As a further internal control for the adoptive transfer experiment, a proportion of labelled monocytes were used for an *in vitro* invasion assay. Monocytes pre-treated with MMP-14 blocking antibody demonstrated a 1.9-fold reduced ability to invade a synthetic matrix compared with IgG pre-treated, control monocytes (*P* < 0.05; see Supplementary material online, *Figure S7B*). Collectively, these data strongly suggest a predominant role for MMP-14 in mediating monocyte recruitment and macrophage accumulation within established atherosclerotic plaques, highlighting both MMP-14 and its inhibitor, TIMP-2, as potential therapeutic avenues for the treatment of unstable atherosclerotic lesions.

### 3.6 TIMP-2 diminishes macrophage apoptosis and retards MMP-14-dependent N-cadherin cleavage

We observed that atherosclerotic plaques from *TIMP-2^−/−^ Apoe^−/−^* exhibited heightened frequencies of macrophage apoptosis compared with *Apoe^−/−^* controls (*Table 1* and *Figure 1*). In an attempt to elucidate the underlying mechanisms by which TIMP-2 impedes macrophage apoptosis, macrophages from *TIMP-2^−/−^* and *TIMP-2^+/+^* control mice were subjected to LPS-induced apoptosis. In accordance with our histolological findings, the absence of TIMP-2 increased macrophage apoptosis by 6-fold (8.2 ± 4.4 vs. 49.4 ± 3.3%; *P* ≤ 0.001; *n* = 5). Consequently, western blotting for the pro-survival protein N-cadherin was conducted to gain mechanistic insight into the anti-apoptotic effect of TIMP-2, as we have previously demonstrated that MMP-mediated cleavage of N-cadherin can induce macrophages apoptosis.^9,32^ We demonstrate that GM-CSF stimulation of macrophages, which we have previously shown induces MMP-14 activity and apoptotic frequencies,^16^ induces the generation of cleaved N-cadherin expression in macrophages, but this is reversed in the presence of exogenous TIMP-2 (*P* < 0.01; see Supplementary material online, *Figure S8*). Moreover, addition of an MMP-14-blocking antibody alone or in combination with exogenous TIMP-2 negated the formation of the cleaved fragment of N-cadherin observed in the presence of GM-CSF (*P* < 0.05 and *P* < 0.01, respectively; see Supplementary material online, *Figure S8*). The additive effect may suggest that other proteases capable of N-cadherin cleavage, such as MMP-12,^9^ are amenable to TIMP-2 inhibition. Indeed, it has recently been demonstrated that TIMP-2 can retard MMP-12 activity.^33^

## 4. Discussion

It is considered that protease inhibitors most probably play an important protective role, during atherosclerosis, although there is no consensus about their relative importance. Our current findings clearly demonstrate disparate roles for TIMP-1 and TIMP-2 in plaque evolution. We show that loss of TIMP-1 expression has no major effect on plaque size or composition in the brachiocephalic artery of *Apoe*^−/−^ mice, except for reducing smooth muscle cell content. In contrast, mice with no functional TIMP-2 gene have more complex plaques and exhibit several compositional changes associated with increased plaque vulnerability compared with TIMP-1-deficient mice, as well as *Apoe*^−/−^ wild-type controls. Similarly, while TIMP-1 does not substantially regulate monocyte/macrophage invasion, TIMP-2 exerts a major inhibitory role. These findings strongly imply a prominent role for endogenous levels of TIMP-2 in the reduction of atherosclerotic plaque development and progression, particularly through the retardation of monocyte/macrophage accumulation, presumably through MMP inhibition. Indeed, we show for the first time that monocyte/macrophage accumulation within established mouse atherosclerotic plaques is MMP-14 dependent. Moreover, MMP-14-mediated monocyte invasion *in vitro* and *in vivo* is restricted by endogenous levels of TIMP-2. The continual accumulation of monocyte/macrophages with their associated heightened proteolytic repertoire is considered fundamental to the development, progression, and destabilization of atherosclerotic plaques.^34,35^

The minor effects of TIMP-1 knockout we observed on plaque size and composition are in broad agreement with previous findings.^21,36,37^ In one study, modest increases in macrophage and lipid content were reported in lesions at the aortic root of *TIMP-1^−/−^ Apoe^−/−^* mice relative to *Apoe^−/−^* controls, despite a paradoxical reduction in plaque size.^38^ The discrepancy with our findings could be owing to minor differences in strain background and diet in the two studies. Overexpression of TIMP-1 also reduced atherosclerotic plaque size at the aortic root of *Apoe^−/−^* mice in one study,^39^ but this effect was not replicated in another.^37^ In neither of these overexpression studies were effects on plaque composition quantified. However, the study from Lemaitre and colleagues did suggest that plaque smooth muscle cell numbers were diminished in *TIMP-1^−/−^ Apoe^−/−^* mice,^36^ which is in agreement with our current findings. It is plausible that TIMP-1 promotes cell survival through the inhibition of MMP-7. TIMP-1 has been shown to potently retard MMP-7 activity,^40^ while MMP-7 can generate active soluble FasLigand (FasL),^40^ an effective inducer of smooth muscle cell apoptosis^41^ Furthermore, we have shown previously that MMP-7 can cleave N-cadherin and promote smooth muscle cell apoptosis.^42^ Accordingly, we observed reduced frequencies of smooth muscle cell apoptosis in plaques from *MMP-7^−/−^ Apoe^−/−^* mice,^42^ associated with increased smooth muscle cell number, but no discernible effects on plaque area or other compositional elements,^43^ consistent with our observations in *TIMP-1^−/−^ Apoe^−/−^* mice. Taken together, the studies imply a small role at best for TIMP-1 in plaque evolution in mice.

The dramatic shift in brachiocephalic plaque composition towards vulnerability caused by deletion of TIMP-2 accords with a previous study, which intimated that overexpression of exogenous TIMP-2 favourably affects plaque phenotype, possibly through modulation of macrophage behaviour.^21^ However, the potential beneficial anti-proteolytic effects afforded from elevated exogenous TIMP-2 levels on murine atherosclerosis were not fully elucidated, although the involvement of gelatinolytic MMPs were implied by *in situ* zymography. Nonetheless, overall TIMP-2 seems to be a much more important determinant of plaque stability than TIMP-1. It must be noted that the number of buried layers within atherosclerotic lesions were not different between TIMP-1- and TIMP-2-deficient mice, although a difference was observed between the TIMP-1 and TIMP-2 wild-type controls. We believe this is as a result of slight genetic drift between the founder mice, as we have previously observed differences in the number of intra-plaque buried fibrous layers between MMP wild-type mice generated for knockout mouse studies.^43^

Despite similar structural properties, functional differences between TIMP-1 and TIMP-2 have been reported.^44^ For example, TIMP-1 displays poor inhibitory capacity towards membrane-type (MT) MMPs, whereas these are completely inhibited by TIMP-2.^20^ The striking differences observed between TIMP-1 and TIMP-2 deficiency are therefore most easily explained by disparate inhibitory effects on specific MMPs. Several reviews have highlighted the different roles of specific MMPs that emerge from knockout studies in the same *Apoe*^−/−^ background.^17,45^ For example, we previously demonstrated that MMP-3, MMP-7, MMP-9, and MMP-12 exert highly divergent effects on atherosclerotic plaque development, particularly cellular composition, in mice studied under very similar conditions.^43^ MMP-14, also known as MT1-MMP, has been linked to atherosclerosis progression and plaque vulnerability in mice^15,16^ owing to its ability to deplete collagen. MMP-14 has also been observed in human plaques where its abundance associates with markers of greater vulnerability.^13,46^ Additionally, MMP-14 has been shown to cluster at the lamellipodia of migrating monocytes, and inhibition of MMP-14 activity (either by neutralizing antibody or gene silencing) retards *in vitro* monocyte transmigration through activated endothelium.^10,11^ Previous studies have also demonstrated that broad spectrum MMP inhibitors or TIMP-2 suppress mononuclear cell transmigration *in vitro* and *in vivo*, whereas TIMP-1 is ineffective.^11,21,47^ Accordingly, we hypothesized that the loss of TIMP-2 in mice might result in heightened monocyte/macrophage MMP-14 activity, facilitating macrophage accumulation within atherosclerotic lesions and thereby provoking the detrimental shift in plaque composition.

Consistent with this hypothesis, our present results demonstrate for the first time that only LyC6^lo^ CCR2^lo^ CX3CR1^hi^ monocytes constitutively express cell-surface MMP-14. Indeed, our findings show that MMP-14 may serve as an additional marker of the LyC6^lo^ CCR2^lo^ CX3CR1^hi^ monocyte subset that performs a patrolling role in early responses to vascular^29^ and myocardial injury.^30^ However, upon adherence, the majority of monocytes, including the LyC6^hi^ CCR2^hi^ CX3CR1^lo^ subset, acquire activated and cell-membrane-localized MMP-14, which is therefore available to facilitate cell invasion in response to MCP-1 and/or fractalkine chemo-attraction.^11^ Hence, although divergent monocyte subsets may display disparate levels of other MMPs,^7^ adherence modulates MMP-14 expression irrespective of monocyte subset. Our *in vitro* and *in vivo* data yield strong evidence that MMP-14 facilitates the invasion of adherent monocyte/macrophages in a TIMP-2-dependent manner. Indeed we demonstrate for the first time that *in vivo* macrophage invasion is augmented in the absence of TIMP-2, but not TIMP-1. This novel finding supports our previous studies, in which exogenous TIMP-2 expression retarded *in vitro* monocyte/macrophage invasion.^21^

The present study also demonstrates a specific effect of MMP-14 activity on monocyte/macrophage accumulation within murine atherosclerotic lesions. We therefore postulate that heightened MMP-14 activity afforded by loss of TIMP-2 underpins the increased macrophage content observed within brachiocephalic plaques of *TIMP-2^−/−^ Apoe^−/−^* mice when matched to *TIMP-2^+/+^ Apoe^−/−^* controls or *TIMP-1^−/−^ Apoe^−/−^* mice. Supporting findings were observed in a mouse model of myocardial infarction, where TIMP-2-deficient mice exhibit increased monocyte/macrophage infiltration alongside heightened MMP-14 expression and activity, promoting adverse disease progression compared with wild-type controls.^48^ Also, the observation that increased elastin fragmentation alongside macrophage infiltration was more evident in atherosclerotic plaques of TIMP-2-deficient mice, implies a protective role for TIMP-2 in the pathogenesis of aortic aneurysms. Finally, increased monocyte/macrophage accumulation within atherosclerotic plaques is related with heightened macrophage proliferation^5^ and augmented frequencies of apoptosis.^49^ Hence, we can postulate that loss of TIMP-2 and associated increased MMP-14 activity promotes monocyte/macrophage accumulation within developing plaques, and consequent macrophage apoptosis (through TIMP-2-dependent MMP cleavage of N-cadherin for example) induces further monocyte recruitment via the release of numerous chemotactic factors.^49^Ensuing plaque progression associated with necrotic core expansion has been shown to favour heightened local macrophage proliferation.^5^ This tenet is in accordance with our findings where TIMP-2 deficiency results in advanced plaques exhibiting increased macrophage number alongside amplified rates of macrophage proliferation and augmented frequencies of macrophage apoptosis.

We cannot discount the inhibitory effects of TIMP-2 on other MMPs, particularly type I MT-MMPs, which might contribute to our observed effects. Indeed recent studies^50,51^ have demonstrated the expression of other type I MT-MMP family members (MMP-15, −16, and −24) in human atherosclerotic plaques, although their direct role in monocyte recruitment and plaque progression await the appropriate experimentation. However, our findings that MMP-14 is substantially greater expressed by monocytes and macrophages imply that the other type I MT-MMPs play a minor role. Additionally the gelatinases MMP-2 and −9 also appear to play negligible roles in monocyte/macrophage gelatinolytic activity and associated invasion. Furthermore, we previously demonstrated that MMP-14 is the most abundant MMP in activated macrophages.^52^ Indeed, the fact that we were able to inhibit monocyte recruitment *in vivo* with a specific antibody against MMP-14 demonstrates that it plays an indispensable role. It is also possible that the beneficial effects TIMP-2 exert on atherosclerosis may be through the suppression of numerous proteolytic substrates and the modulation of other resident cell types within atherosclerotic lesions. While atherosclerotic plaque smooth muscle cells and endothelial cells have been shown to express TIMP-2, endothelial cell TIMP-2 expression is decreased compared with healthy vessels.^3^ MMP-14 is also expressed by plaque smooth muscle cells and endothelial cells, but to a lesser degree than macrophages.^53,13^ Moreover, with regard to the contribution of endothelial cell-derived TIMP-2 and/or MMP-14 to monocyte migration, Sithu and colleagues^54^ demonstrated that either endothelial cell or monocyte-derived TIMP-2 could reduce monocyte transmigration, while in support of our own findings TIMP-1 could not. Similarly, MMP-14 in endothelial cells or monocytes promoted transmigration (via ICAM-1 cleavage), which could be blocked by TIMP-2, but not TIMP-1. Accordingly, effects on endothelial cell-derived TIMP-2 and/or MMP-14 may also in part contribute to the observed effects *in vivo*.

In conclusion, our current findings in double knockout mice highlight varying roles for different members of the TIMP family in atherosclerotic plaque development and progression. The fact that TIMP-2 plays a protective role while that of TIMP-1 is minimal, supports the tenet that differing MMPs impart divergent effects on atherosclerosis.^43^ Moreover, we provide strong evidence for a detrimental role of monocyte/macrophage MMP-14 expression/activity in atherosclerotic plaque development and progression, and illustrate it as a therapeutic target. In association, promoting TIMP-2 expression may serve as an effective avenue to retard monocyte recruitment to sites of inflammation such as atherosclerotic plaques, while additionally perturbing intra-plaque MMP activity and increasing lesion stability.

## Supplementary material

Supplementary material is available at *Cardiovascular Research* online.

## Funding

This work was supported by grants from the British Heart Foundation (FS/07/053/24069 and FS/09/010/26488, both to J.L.J.) and support from the National Institute for Health Research Bristol Biomedical Research Unit in Cardiovascular Medicine. Funding to pay the Open Access publication charges for this article was provided by…
