# Corrigendum

**DOI:** 10.1093/cvr/cvw202

**Published:** 2016-10-19

**Corrigendum to:** Differential effects of tissue inhibitor of metalloproteinase (TIMP)-1 and TIMP-2 on atherosclerosis and monocyte/macrophage invasion [*Cardiovasc Res* 2016; **109** (2): 318–330]

Dr. Jackson has asked for his name to be removed. He contributed to elements of the reported studies during the initial submission but he did not contribute to the revision and the final manuscript was submitted without his formal approval. This has now been corrected online via post-production correction.

